# Significance of decoy receptor 3 in sera of hepatocellular carcinoma patients

**DOI:** 10.3109/03009734.2010.516410

**Published:** 2010-10-27

**Authors:** Meisongzhu Yang, Gang Chen, Yiwu Dang, Dianzhong Luo

**Affiliations:** ^1^Department of Pathology and Pathophysiology, Medical College of Jishou University, Jishou 416000, People's Republic ofChina; ^2^Department of Pathology, Guangxi Medical University, Nanning 530021, People's Republic ofChina

**Keywords:** Decoy receptor 3 (DcR3), enzyme-linked immunosorbent assay (ELISA), hepatocellular carcinoma (HCC), immunohistochemistry (IHC)

## Abstract

**Objective:**

Decoy receptor 3 (DcR3), a member of the tumor necrosis factor receptor superfamily, is amplified and over-expressed in various cancers. The objective of the present study was to investigate the concentration of DcR3 in sera of hepatocellular carcinoma (HCC) patients and its clinical significance.

**Methods:**

Serum concentrations of DcR3 were measured by enzyme-linked immunosorbent assay (ELISA) in 67 patients with HCC, 8 with liver cirrhosis, 17 with cholecystitis, and in 28 healthy individuals. Immunohistochemistry was employed to access protein expression of DcR3 in the corresponding HCC tissues.

**Results:**

Serum concentrations of DcR3 in patients with HCC or cirrhosis were significantly higher than in healthy individuals (*P* < 0.01). Moreover, serum concentrations of DcR3 in HCC patients were associated with TNM stage, para-cirrhosis, capsular infiltration, and metastasis or recurrence of disease (*P* < 0.05). There was a positive correlation between the serum concentration of DcR3 and protein expression in HCC tissues (*r* = 0.472, *P* < 0.01).

**Conclusions:**

The high serum concentration of DcR3 might play a certain role in pathogenesis, progress, and metastasis of HCC. Moreover, DcR3 might serve as a valuable molecular indicator in early diagnosis and contribute to predicting the clinical outcome in HCC patients.

## Introduction

Hepatocellular carcinoma (HCC) is the fifth most common cause of cancer deaths in the world with an increasing incidence (1–3). Although the prognosis of HCC patients has marginally been improved over the last two decades, the 5-year survival rate still remains around 5% (4,5). Presently, standard surveillance includes a combination of 6-monthly abdominal ultrasound scans and serum alphafetoprotein (AFP) measurements, but this strategy cannot reliably detect early liver tumors. Serum AFP may also be elevated in cirrhosis, especially with viral hepatitis, in the absence of HCC. The differential diagnosis of elevated serum AFP concentrations also includes gastric, biliary, and pancreatic cancers and germ cell tumors (6). The relatively suboptimal sensitivity of conventional serum AFP as a marker has led to a search for more sensitive and specific markers (7). It has been reported that decoy receptor 3 (DcR3)/TR6/M68 was over-expressed in various malignant tumor tissues as regards both mRNA and protein levels (8–17). In addition, serum concentrations of DcR3 were elevated in cancers of the stomach, liver, pancreas, gall-bladder, colon, thyroid, lung, kidney, breast, and ovary (8–12) as well as in gliomas (17). Our group previously studied the protein expression of DcR3 in HCC microarrays and found that the positive rate of DcR3 in HCC tissues was significantly higher than that in para-cancerous, cirrhosis, and normal liver. DcR3 expression in HCC was correlated with clinical TNM stages, the status of metastasis or recurrence of disease, serum AFP concentration, portal vein tumor emboli, capsular infiltration, and multiple tumor nodes (14). Compared to the evaluation of DcR3 protein detection, Wu et al. (8) established an enzyme-linked immunosorbent assay (ELISA) to measure DcR3 serum concentrations in HCC, and 74.3% (26/35) of the patients were found to be DcR3-positive (>20 pg/mL). Likewise, liver cirrhosis was found to be a condition with elevated serum DcR3 concentrations. Immunohistochemistry was performed to examine the DcR3 expression in HCC and cirrhosis tissues by Wu et al. (8). However, only a small number of samples were examined. To our knowledge, no studies have evaluated the associations between DcR3 serum concentrations and protein expression and clinical parameters in the same HCC patient population. Therefore, we investigated the relationship between DcR3 serum concentrations and its protein expression in HCC tumor tissues and clinicopathological parameters.

## Patients and methods

### Patients

Serum samples were collected at the First Affiliated Hospital, Guangxi Medical University, China, between September 2006 and September 2007 from patients with HCC (*n* = 67), cirrhosis (*n* = 8), or cholecystitis (*n* = 17) and also from healthy controls (*n* = 28). Clinical information was obtained from medical records. The age of the HCC patients ranged from 29 to 74 years, with a mean of 48 years. Among the 67 patients, 58 were male and 9 female. HBV infection status was based on hepatitis B surface antigen (HBsAg). Fifty-one patients were HBsAg-positive, and ten were found to be anti-HCV-positive. There were six anti-HCV-positive cases that were co-infected with HBV, and six cases were both HBsAg-negative and anti-HCV-negative. The histopathologic diagnoses were made according to the World Health Organization international histological classification of HCC: well differentiated (*n* = 16), moderately differentiated (*n* = 37), or poorly differentiated (*n* = 14). According to the clinical tumor–node–metastasis (TNM) standard, their clinical stages were stage I (*n* = 26), stage II (*n* = 11), stage III (*n* = 19), and stage IV (*n* = 11). All 67 HCC patients were followed up for 20 months by measurements of tumor markers (AFP) and ultrasound (US), as well as helical computed tomographic (CT) scan. Forty cases had metastasis or recurrence of disease, 27 cases did not. Forty-four cases had para-cirrhosis. Sixty-four samples of HCC tissues were collected from hepatectomies. All 64 cases were initial hepatectomies in order to avoid the secondary changes of healing *post* biopsy. All the 67 HCC patients had never received any radiation therapy or chemotherapy. Diagnosis and classification were based on histology or cytology by the same pathologist. Among eight cirrhotic samples, six were male, two female. The range of age was between 28 and 60 years, with a mean of 44 years. In the cholecystitis group, nine were male, eight female. The age ranged from 25 to 79 years, with a mean of 55 years. After formation of a clot, sera were collected and centrifuged at 1,000 rpm for 20 min. The supernatant was carefully aspirated and stored at −80°C until analysis. The study protocol was approved by Guangxi Medical University Ethics Committee. Written informed consent to use the samples for research was obtained from the patients and clinicians.

## Methods

### Measurements of serum DcR3 concentrations

To measure DcR3 concentrations in sera, a human DcR3 ELISA kit (Bender Med Systems, Vienna, Austria) was used according to the manufacturer's protocol. Briefly, 100 μL of each sample was incubated in duplicate in microplates coated with anti-DcR3 monoclonal antibody for 2 h. Following incubation, biotin-conjugate anti-DcR3 monoclonal antibody was added and incubated as a primary antibody for 2 h. After the microplate had been washed, streptavidin-horseradish peroxidase (HRP) was added and incubated for 1 h. After washing off any unbound streptavidin-HRP, tetramethylbenzidine was added as a substrate, and the absorbance was measured at 450 nm after 20 min in a microplate reader (BioTek Instruments, Burlington, VT, USA). The absorbance of each sample was plotted against a standard curve produced by serial dilutions of recombinant human DcR3-Fc in duplicate. The concentrations of DcR3 in sera were calculated by logarithmic analysis. All samples with an absorbance of less than zero according to the standard curve were discarded from the final analysis. A concentration higher than 122.22 pg/mL was set as the positive level according to the DcR3 average level in the normal control group in our study.

### Immunohistochemistry

The procedure employed has previously been described (14). Sections of gastric carcinoma tissue that proved to be positive for DcR3 were used as positive controls. The primary antibody was replaced by phosphate buffer solution for negative controls. The positive signal for DCR3 appeared as yellow-brown in the cytoplasm of the cells using 3,3′-diaminobenzidine. One or two of the most representative sections from each case were chosen and stained with a rabbit polyclonal antibody against DcR3 (DcR3<H-130>:sc-25464, 1:300 dilution) from Santa Cruz Biotechnology, Inc., Heidelberg, Germany, which is raised against amino acids 171–300 of DcR3 of human origin. One hundred cells from five representative areas from each case were counted. Staining results were evaluated according to immunodetection of stain intensity and number of positive cells by two pathologists (G.C. and D.L.), who discussed each case until they reached a consensus. Staining intensity was up to the standard of the relative staining intensity of most cells. The degree of staining was subdivided as follows: the staining intensity could range from 0 to 3 (0 = no staining; 1 = yellow or light brown, weak staining; 2 = brown, strong staining; and 3 = dark brown, intense staining), and the positive cells in the observed liver tissues ranged from 0 to 3 in percentage (0, no staining; 1, <30%, often focal or fine granular; 2, 30%–70%, linear or cluster; and 3 >70%, diffuse). Samples were scored by their summation: 0–1 (-); 2–3 (+); 4 (++); 5–6 (+++). Any staining score ≥2 (+) was considered as positive expression.

### Statistical analyses

All data from ELISA were expressed as mean ± standard deviation (SD). For normally distributed data, the two-tailed Student's *t* test and the Mann–Whitney test were used for comparisons between the two groups with SPSS 17.0 software for Windows (Munich, Germany). Correlations were calculated by Pearson chi-square test. In all cases, a *P* value of less than 0.05 was considered statistically significant.

## Results

### Comparison of serum DcR3 concentrations and DcR3 expression in different diseases

The serum concentration of DcR3 in the HCC patients was higher than in the cholecystitis patients and in the healthy controls (*P* < 0.01). Likewise, in the cirrhosis patients the DcR3 serum concentration was higher than that in the healthy controls (*P* < 0.01, Table I). Quite in contrast, DcR3 serum concentrations were not elevated in cholecystitis patients.

**Table I.** Serum concentrations of DcR3 in different diseases.

			*P* compared with		
Diseases	DcR3 serum level (pg/mL)	DcR3 expression rate	Cirrhosis	Cholecystitis	Healthy controls
HCC	197.07 ± 90.34	51/67 (76%)	0.094	0.002	0.005
Cirrhosis	179.81 ± 102.74	5/8 (62%)	—	0.083	0.003
Cholecystitis	101.59 ± 24.51	3/17 (18%)	—	—	0.102
Healthy controls	96.69 ± 16.05	1/28 (4%)	—	—	—

### Relationship between serum DcR3 level of HCC and clinicopathological parameters

Serum concentrations of DcR3 in patients with clinical TNM stage III and IV were higher than in patients at stages I and II. Serum concentrations of DcR3 in patients with para-cirrhosis, capsular infiltration, and metastasis or recurrence of disease were significantly higher than in those without (*P* < 0.05, Table II).

**Table II.** Relationship between serum DcR3 concentrations and clinicopathological parameters in hepatocellular carcinoma (HCC).

HCC clinicopathological parameters		Serum DcR3 level (pg/mL)	Serum DcR3 expression rate	*P*
Age	≥50	207.90 ± 82.76	25/30 (83%)	0.212
	<50	189.76 ± 95.44	26/37 (70%)	
Gender	male	206.18 ± 92.69	47/58 (81%)	0.048
	female	138.35 ± 40.37	4/9 (44%)	
HBV	positive	221.34 ± 64.34	45/51 (88%)	0.296
	negative	198.14 ± 45.13	6/16 (38%)	
HCV	positive	208.73 ± 45.63	8/10 (80%)	0.187
	negative	178.63 ± 61.04	43/57 (75%)	
Differentiation	well and moderately	187.89 ± 88.45	41/53 (77%)	0.106
	poorly	231.84 ± 92.13	10/14 (71%)	
Clinical TNM stage	I–II	160.76 ± 62.57	26/37 (70%)	0.014
	III–IV	224.78 ± 98.85	25/30 (83%)	
Para-cirrhosis	yes	212.76 ± 92.60	37/44 (84%)	0.005
	no	150.94 ± 66.11	14/23 (61%)	
Tumor capsular infiltration	no capsular or capsular infiltration	219.52 ± 95.59	32/37 (86%)	0.015
	no capsular infiltration	167.66 ± 74.70	19/30 (63%)	
Portal vein tumor embolus	yes	198.26 ± 84.80	9/11 (82%)	0.944
	no	196.56 ± 93.48	42/56 (75%)	
Tumor nodes	multi	207.34 ± 94.55	20/25 (80%)	0.477
	single	190.96 ± 88.33	31/42 (74%)	
Tumor size (cm)	≥5	207.05 ± 89.54	35/43 (81%)	0.304
	<5	184.00 ± 91.26	16/24 (67%)	
AFP (μg/L)	≥400	213.10 ± 90.42	23/28 (82%)	0.327
	<400	185.56 ± 89.66	28/39 (72%)	
Metastasis or recurrence	yes	215.04 ± 93.63	34/40 (85%)	0.02
	no	164.88 ± 75.66	17/27 (63%)	

### Correlation between the concentration of serum DcR3 and expression of DcR3 protein in HCC tissues

**Figure 1.**
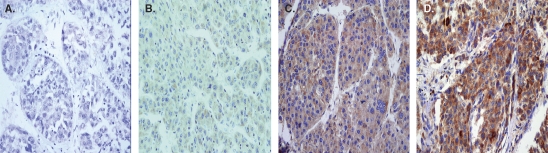
Expression of DcR3 protein in hepatocellular carcinoma tissues. DcR3 protein was detected by immunohistochemistry in hepatocellular carcinoma (HCC) tissues. (A: DcR3 negative; B: positive+; C: ++; D: +++). The signal was localized within the cytoplasm of tumor cells in HCC tissues (×400).

Sixty-four tissues from the total 67 cases were studied with immunohistochemistry to detect the expression of DcR3 protein (Figure 1). There was a positive correlation between the concentrations of DcR3 in sera and protein expression in HCC tissues (*r* = 0.472, *P* < 0.01). The rate of DcR3 protein expression in HCC tissues was 61% (39/64), which was lower than the corresponding rate of DcR3 serum-positive (higher than 122.22 pg/mL) patients (77%, 49/64, *P* < 0.05, Table III). Likewise, 35 cases among the 39 DcR3 protein expression-positive patients were all detected to be DcR3 serum-positive. Fourteen of the serum DcR3-positives were found to be DcR3 protein expression-negative.

**Table III.** Correlation between the level of serum DcR3 and the expression of DcR3 protein in hepatocellular carcinoma (HCC) tissues.

		Expression of DcR3 protein in HCC tissues			
Level of serum DcR3 (pg/mL)	*n*	-	+	++	+++
Negative (0–122.22)	15	11	4	0	0
Low level (122.23–149.99)	16	6	5	4	1
Moderate level (150.00–299.99)	20	6	8	4	2
High level (≥300.00)	13	2	4	2	5

### Overall diagnostic effectiveness of mutual detection of AFP and DcR3

The sensitivities of single detection of AFP and DcR3 were 82% and 76%, respectively (detailed data not shown). The sensitivity of combined detection of AFP and DcR3 increased to 93% (detailed data not shown).

## Discussion

Human DcR3 is a soluble receptor that is mostly expressed in many different classes of tumors. DcR3 is short of the transmembrane domain of conventional Tumor Necrosis Factor Receptors (TNFRs) and is thus believed to be a secreted protein (16). In the present study, we determined the serum DcR3 concentrations in 67 HCC patients, and 51 cases were positive (76%), significantly higher than that of cholecystitis and of healthy controls. The present data confirmed the results of Wu et al. (8). This confirmation indicates that serum concentrations of DcR3 in HCC are much higher than in non-tumor controls. Our results also showed that serum DcR3 concentrations were elevated in cirrhosis patients. This finding was consistent with that of Wu et al. (8), who reported elevated serum concentrations of DcR3 in five cirrhosis cases. In our previous studies we also found over-expression of DcR3 in cirrhotic tissues (14). Our results together with the data of Wu et al. suggest that there may be a link between DcR3 expression and formation of cirrhosis. Moreover, in HCC patients, DcR3 serum concentrations in the para-cirrhosis-positive group were higher than in the para-cirrhosis-negative group. Forty-four patients in the total of 67 (66%) had para-cirrhosis (Table II). Among these 44 patients, there were 37 (84%) who were serum DcR3-positive. Albeit entirely speculatively, it might well be that a part of their serum DcR3 derived from the para-cirrhosis.

It is known that HCC can result from pre-cancerous liver disease, for instance cirrhosis. However, the molecular mechanisms in such a process remain unclear. The high serum concentrations of DcR3 in the cirrhosis patients and in the para-cirrhosis HCC patients in the present study prompted us to formulate a hypothesis concerning the potential role of DcR3 in hepatocarcinogenesis. However, further studies have to be carried out to prove the hypothesis and investigate the value and mechanism of DcR3 in liver cirrhosis and tumorigenesis of HCC.

To our knowledge, this is the first study investigating a possible relationship between serum concentrations and clinicopathological parameters of patients in HCC. Another interesting observation in the present study was that the serum concentration of DcR3 directly correlated with para-cirrhosis, capsular infiltration, and metastasis or recurrence of disease (*P* < 0.05), revealing an obvious relation between DcR3 and HCC malignant characteristics. As a rule, the capsular infiltration and metastasis reflect tumor growth and tumor invasive capability.

To date, to our knowledge there has been no study reporting the association between the serum DcR3 concentration and the protein DcR3 expression in HCC tissues. To answer this question, we performed immunohistochemistry in 64 among the 67 HCC patients to measure DcR3 protein expression and compared it with the corresponding serum DcR3 concentration. Our results showed that there was a positive correlation between the serum DcR3 concentration and the protein DcR3 expression in HCC. However, the serum DcR3-positive rate (77%) was higher than the corresponding tissue DcR3 protein expression rate (39%). This was due to 14 cases of negative expression of DcR3 protein in patients, who were all that serum-positive. The variation between serum and tissue DcR3 levels might result from a difference in the sensitivity of the detecting assays (ELISA versus immunohistochemistry). Thus, using ELISA to estimate serum DcR3 concentrations seems more advantageous in clinical application.

Taken together, our study demonstrates that the measurements of serum DcR3 concentrations might become of value for clinical early diagnosis, prognosis prediction, and curative effects in HCC.

## References

[1] El-Serag HB, Rudolph KL (2007). Hepatocellular carcinoma: epidemiology and molecular carcinogenesis. Gastroenterology.

[2] Jaeck D, Bachellier P, Oussoultzoglou E, Weber JC, Wolf P (2004). Surgical resection of hepatocellular carcinoma. Post-operative outcome and long-term results in Europe: an overview. Liver Transpl.

[3] Orito E, Mizokami M (2007). Differences of HBV genotypes and hepatocellular carcinoma in Asian countries. Hepatol Res.

[4] El-Serag HB, Mason AC, Key C (2001). Trends in survival of patients with hepatocellular carcinoma between 1977 and 1996 in the United States. Hepatology.

[5] Nagai H, Sumino Y (2008). Therapeutic strategy of advanced hepatocellular carcinoma by using combined intra-arterial chemotherapy. Recent Patents Anticancer Drug Discov.

[6] Colombo M (2001). Screening for cancer in viral hepatitis. Clin Liver Dis.

[7] Sherman M (2001). Alphafetoprotein: An obituary. J Hepatol.

[8] Wu Y, Han B, Sheng H, Lin M, Moore PA, Zhang J (2003). Clinical significance of detecting elevated serum DcR3/TR6/M68 in malignant tumor patients. Int J Cancer.

[9] Macher-Goeppinger S, Aulmann S, Wagener N, Funke B, Tagscherer KE, Haferkamp A (2008). Decoy receptor 3 is a prognostic factor in renal cell cancer. Neoplasia.

[10] Simon I, Liu Y, Krall KL, Urban N, Wolfert RL, Kim NW (2007). Evaluation of the novel serum markers B7-H4, Spondin 2, and DcR3 for diagnosis and early detection of ovarian cancer. Gynecol Oncol.

[11] Connor JP, Felder M (2008). Ascites from epithelial ovarian cancer contain high levels of functional decoy receptor 3 (DcR3) and is associated with platinum resistance. Gynecol Oncol.

[12] Anderson GL, McIntosh M, Wu L, Barnett M, Goodman G, Thorpe JD (2010). Assessing lead time of selected ovarian cancer biomarkers: a nested case-control study. J Natl Cancer Inst.

[13] Chen G, Luo D (2008). Over-expression of decoy receptor 3 in gastric precancerous lesions and carcinoma. Ups J Med Sci.

[14] Chen G, Luo D (2008). Expression of decoy receptor 3 in liver tissue microarrays. Natl Med J India.

[15] Shen HW, Gao SL, Wu YL, Peng SY (2005). Overexpression of decoy receptor 3 in hepatocellular carcinoma and its association with resistance to Fas ligand-mediated apoptosis. World J Gastroenterol.

[16] Pitti RM, Marsters SA, Lawrence DA, Roy M, Kischkel FC, Dowd P (1998). Genomic amplification of a decoy receptor for Fas ligand in lung and colon cancer. Nature.

[17] Hwang SL, Lin CL, Cheng CY, Lin FA, Lieu AS, Howng SL (2004). Serum concentration of soluble decoy receptor 3 in glioma patients before and after surgery. Kaohsiung J Med Sci.

